# Phytosterols: Extraction Methods, Analytical Techniques, and Biological Activity

**DOI:** 10.3390/molecules30122488

**Published:** 2025-06-06

**Authors:** Byoung-Hoon Yoon, Van-Long Truong, Woo-Sik Jeong

**Affiliations:** 1 School of Food Science & Biotechnology, College of Agriculture and Life Sciences, Kyungpook National University, Daegu 41566, Republic of Korea; byounghoon97@naver.com; 2 Food and Bio-Industry Research Institute, School of Food Science & Biotechnology, College of Agriculture and Life Sciences, Kyungpook National University, Daegu 41566, Republic of Korea; truonglongpro@gmail.com

**Keywords:** phytosterols, extraction methods, analytical techniques, biological activities

## Abstract

Phytosterols, essential components of plant cell membranes, are abundant in fruits, vegetables, nuts, and seeds. Among them, β-sitosterol, campesterol, and stigmasterol have drawn significant interest for their well-documented biological activities. This review highlights recent advancements in extraction and analytical methods aimed at improving phytosterol yield, maintaining chemical stability, and reducing environmental impact. These innovative, eco-friendly techniques offer promising alternatives to traditional extraction approaches and hold potential for industrial-scale use. Phytosterols possess various bioactive properties, including antioxidant, anti-inflammatory, chemopreventive, cholesterol-lowering, and neuroprotective effects. Through these mechanisms, dietary phytosterols may help prevent cardiovascular and neurodegenerative diseases, type 2 diabetes, and certain cancers. Recent studies have focused on the identification, isolation, purification, and characterization of phytosterols from diverse food matrices, along with assessing their health benefits. However, the specific molecular pathways responsible for their pharmacological actions remain poorly understood, highlighting the need for further research, particularly in human clinical trials. This review provides a comprehensive overview of the extraction strategies, biological functions, and mechanisms of action of phytosterols, offering valuable insights for developing phytosterol-enriched functional foods and nutraceuticals.

## 1. Introduction

Nutrition is fundamental for human metabolic processes and the overall functioning of physiological systems [1]. Advancements in food processing and distribution technologies have markedly improved the accessibility and diversity of food products. Nevertheless, these processes may also lead to the degradation or alteration of essential nutrients, potentially adversely affecting human health [2,3]. As a result, there is a growing consumer demand for food products that not only meet fundamental nutritional needs but also offer additional health advantages [4,5]. This demand has prompted the rapid advancement of functional food products specifically designed to provide physiological benefits that extend beyond basic nutritional value. Concurrently, consumers are increasingly favoring natural products that enhance health and well-being while minimizing adverse effects. In this context, a wide variety of plant-derived bioactive compounds, including flavonoids, carotenoids, phenolic compounds, alkaloids, and sterols, have been investigated for their biological effects, which encompass anticancer properties, cardiovascular protection, liver protection, anti-obesity effects, and neuroprotection [4,5,6,7,8,9]. These findings underscore the potential of naturally occurring phytochemicals in promoting health and preventing chronic diseases, thereby enhancing their significance in the formulation of functional foods.

Phytosterols, which are lipid-soluble compounds belonging to the triterpene family, are predominantly located within plant cells, where they play a vital role in maintaining membrane integrity and structural stability. To date, more than 250 distinct phytosterol compounds have been identified across various plant species. These bioactive compounds are particularly abundant in vegetable oils, nuts, seeds, and whole grains, with β-sitosterol, campesterol, and stigmasterol being the most prevalent and extensively studied phytosterols.

Phytosterols exhibit a structural similarity to cholesterol, the primary sterol present in animal cells, which is essential for maintaining the integrity of cell membranes. Both cholesterol and phytosterols share a comparable tetracyclic ring structure that constitutes the core framework of sterols. The primary distinction lies in the side chain located at the C-17 position [10,11]. Additional distinctions pertain to the positioning of double bonds, which are typically located between carbon atoms C-5 and C-6 or between C-7 and C-8. Furthermore, variations in the side chain at the C-24 position are also observed. For instance, β-sitosterol and stigmasterol possess an ethyl group at the C-24 position, while campesterol contains a methyl group at the same site [12]. These structural variations influence their biological activity, absorption, and interactions with enzymes and transporters that are integral to sterol metabolism.

**Figure 1:**
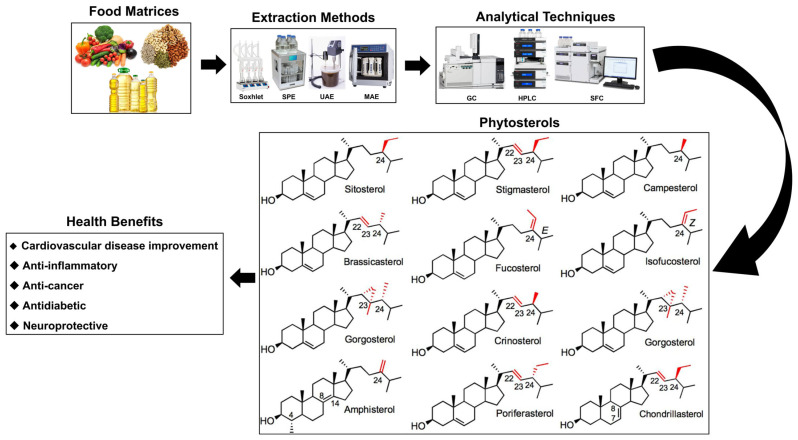
The structures of major phytosterols, along with their extraction and analytical methods, and their roles in biological activity. Red highlights in the chemical structures indicate the positions of double bonds or methyl/ethyl substitutions (typically at C22, C23, and C24), which are key differentiating features among phytosterols. Numbering refers to standard steroid ring carbon positions. SPE, solid-phase extraction; UAE, ultrasonic-assisted extraction; MAE, microwave-assisted extraction; GC, gas chromatography; SFC, supercritical fluid chromatography.

Phytosterols are biologically active compounds that have been acknowledged for their potential to prevent and ameliorate a range of health issues. These compounds are particularly noted for their capacity to reduce cholesterol absorption, as well as their antioxidant, anticancer, anti-inflammatory, and antibacterial properties [12,13,14,15,16]. Furthermore, phytosterols have the potential to impact cardiovascular diseases, anti-inflammation, cancer, diabetes, and neuroprotection [10,11,14,17,18,19]. Humans are unable to synthesize phytosterols endogenously and must obtain them through dietary sources. In Western nations, the typical daily consumption of phytosterols is approximately 200 mg, whereas this intake is generally greater in Asian countries. Research indicates that a daily intake of 1 to 3 g of phytosterols can result in a reduction of about 15% in low-density lipoprotein cholesterol (LDL-C) levels when compared to individuals who do not incorporate these compounds into their diet [20]. This research seeks to provide a comprehensive examination of the methodologies employed in the extraction of phytosterols, the analytical techniques utilized, their therapeutic applications, and their bioavailability (Figure 1).

## 2. Extraction Methods and Analytical Techniques for Phytosterols

Extraction methodologies and analytical techniques constitute fundamental elements of the analytical process. The extraction phase facilitates the isolation of target analytes from the intricate matrices present in food, plant, or biological samples, thereby enabling precise and dependable analysis. By removing impurities during the extraction process, analytes that fall below the detection threshold can be concentrated and subsequently analyzed, thereby enhancing the overall reliability of the findings.

Recent advances in extraction methods aim to reduce solvent use for greater sustainability, while analytical technologies ensure precise quantification and broaden application. Integrating extraction and detection is key to achieving reliable and reproducible results. Table 1 delineates the extraction methods and analytical techniques of phytosterols employed for various plant matrices.

### 2.1. Conventional Extraction Methods

Phytosterols are lipid-soluble, requiring specialized extraction methods. Traditional techniques like Soxhlet extraction and maceration remain widely used for their isolation from complex mixtures.

#### 2.1.1. Solid–Liquid Extraction (SLE)

SLE employs solvents like hexane, ethanol, or methanol, with selection based on solubility, safety, and application needs. It is simple, cost-effective, and equipment-light, with solvent reuse offering added economic benefits [21]. However, SLE poses several challenges: it often requires large volumes of organic solvents, entails prolonged extraction times, and may result in the coextraction of unwanted interfering substances along with phytosterols, thereby raising environmental and selectivity concerns [21].

#### 2.1.2. Soxhlet Extraction

Soxhlet extraction is an efficient method for isolating compounds from solids via continuous solvent circulation. The solvent repeatedly evaporates and condenses, dissolving target compounds and concentrating them in the receiving flask for thorough extraction [22]. In this study, comparing phytosterol extraction from cocoa butter using Soxhlet extraction, ultrasonic-assisted extraction (UAE), and supercritical CO_2_ extraction with ethanol, the total yield of phytosterols was the highest with supercritical extraction at 6441 ± 0.11 µg/g, followed by UAE at 5106 ± 0.02 µg/g, and the lowest yield was obtained using Soxhlet extraction at 4960 ± 0.01 µg/g [23]. Another study compared Soxhlet and supercritical CO_2_ (SC-CO_2_) extraction for obtaining phytosterol-rich oil from Kalahari melon seeds. Soxhlet extraction using petroleum ether yielded 30.5% oil with 431.1 mg/100 g phytosterols, whereas SC-CO_2_ extraction, optimized at 300 bar and 40 °C, produced 78.6% oil with 1063.6 mg/100 g phytosterols, showing superior efficiency [24]. The lower yield from Soxhlet extraction is likely due to prolonged high temperatures, which can degrade heat-sensitive compounds like phytosterols, making it less suitable for thermolabile bioactives [22].

#### 2.1.3. Saponification

Saponification is a common method for extracting phytosterols using alcoholic solutions of strong bases like KOH or NaOH, either at room temperature or heated. After saponification, unsaponifiable components are typically isolated via multiple solvent extractions and solvent evaporation [25,26].

However, saponification, which occurs at elevated temperatures, increases the likelihood of losing heat-sensitive compounds and involves the use of strong acids and alkalis, presenting safety risks for researchers [25]. Moreover, saponification alone is insufficient for maximizing the extraction efficiency of phytosterols. One study, saponification was combined with UAE to physically disrupt the cell walls of edible brown seaweeds, thereby enhancing the release of intracellular phytosterols into ethanol. This combined approach resulted in a high phytosterol extraction yield of 2.642 ± 0.046 mg/g [27]. In another study, a multi-step process—including saponification, extraction, soap removal, crystallization, and recrystallization—was optimized to isolate β-sitosterol from Masson pine tall oil. A mixture of ethanol and water with KOH was used for saponification; n-hexane was employed to extract the unsaponifiable matter, and butanone was used to remove residual soap. The final crystallization steps involved methanol and a methanol/n-hexane mixture, yielding β-sitosterol with 92% purity and an overall yield of 0.53% [28]. In a more recent study, Niger seed oil was saponified using 3.6 N KOH in ethanol, followed by triple liquid–liquid extraction (LLE) with n-hexane to isolate phytosterols. This process yielded a total phytosterol content of 0.96 ± 0.02 mg/g of oil, with β-sitosterol (0.70 ± 0.01 mg/g), campesterol (0.15 ± 0.01 mg/g), and stigmasterol (0.07 ± 0.01 mg/g) identified as the major components [29]. These studies demonstrate that saponification can be effectively integrated into a multi-step workflow or combined with alternative extraction techniques such as UAE to enhance efficiency.

#### 2.1.4. Maceration

Maceration is a simple, cost-effective method suitable for both preliminary and large-scale phytosterol extraction. It involves soaking ground plant material in a solvent at room temperature, with agitation to enhance solute diffusion. After equilibrium is reached, the extract is filtered or centrifuged, and the process may be repeated with fresh solvent to maximize yield. While maceration is considered an appropriate method for extracting heat-sensitive phytosterols, it is often limited by its prolonged extraction time and relatively low efficiency. Notably, the inability to effectively disrupt plant cell walls can significantly constrain the overall extraction yield [26]. In one study, the antioxidant activity and phytochemical composition of 99.5% ethanol extracts from *Pisum sativum* L. and *Cicer arietinum* L. were evaluated using four extraction methods: maceration, Soxhlet, MAE, and UAE. The presence of phytosterol was qualitatively confirmed through the Salkowski reaction and FTIR analysis; however, quantitative profiling of individual sterols was not performed due to the absence of GC-MS or HPLC analysis. Among the methods tested, maceration was deemed the least efficient due to its prolonged extraction time, low yield, and high solvent consumption factors that also raise environmental and economic concerns [30].

### 2.2. Recent Extraction Methods

In recent years, advancements in extraction technologies such as ultrasonic-assisted extraction (UAE), microwave-assisted extraction (MAE), pressurized liquid extraction (PLE), solid-phase extraction (SPE), solid-phase microextraction (SPME), and liquid–liquid extraction (LLE) have been introduced. These methodologies aim to improve extraction efficiency, reduce extraction duration, safeguard heat-sensitive compounds, optimize specific extraction parameters, and facilitate the extraction of various bioactive substances [31,32,33,34,35,36].

#### 2.2.1. Ultrasonic-Assisted Extraction (UAE)

Ultrasonic-assisted extraction (UAE) uses high-frequency sound waves to create cavitation bubbles, whose collapse generates mechanical forces that break plant cell walls, rapidly releasing intracellular compounds into the solvent [26]. The efficiency of UAE depends on factors such as sample moisture, particle size, solvent type, and sonication time. It is a cost-effective method that offers energy savings and improved extraction yields [32]. For instance, the application of UAE for the extraction of *Crambe* seed oil yielded an approximately 20% enhancement in output and a significant reduction in extraction duration when contrasted with conventional extraction methods [37]. In a comparative study, UAE was applied to extract oil from the seeds of *Cucurbita pepo* L., and this was compared to the conventional Soxhlet extraction method using hexane as a solvent. UAE reduced the extraction time from 6 h to 3 h, increased the oil yield by 5.39%, and resulted in a higher total phytosterol content—2017.5 mg per 100 mL of oil, compared to 1657.6 mg obtained using the Soxhlet method [38]. In a recent study, UAE was employed to efficiently extract phytosterols from pumpkin seeds, yielding 95.46 ± 0.06% oil and a phytosterol concentration of 2017.5 ± 100.1 mg/100 mL of oil [39]. Based on these results, the present research also adopted UAE to achieve high-efficiency phytosterol extraction. This technique is considered practical due to its advantages in reducing extraction time, conserving energy, and preserving product quality. In another study, three extraction methods (maceration, UAE, MAE) were compared for isolating phytosterol-rich oil from Himalayan walnuts. Among them, UAE demonstrated the highest performance. Using *n*-hexane as a solvent and relying on ultrasonic cavitation to effectively disrupt cell walls, UAE achieved the highest oil yield (63.68%) along with the highest concentrations of β-sitosterol (441.63 mg/kg) and brassicasterol (84.64 mg/kg) [40]. Moreover, the oil extracted via UAE exhibited lower acid and peroxide values, indicating enhanced oxidative stability. These findings emphasize UAE as a highly efficient extraction method that not only enhances phytosterol content but also improves the overall quality of the oil, underscoring its potential for producing functional plant-based oils.

#### 2.2.2. Microwave-Assisted Extraction (MAE)

MAE is an efficient method for extracting bioactive compounds, using ion conduction and dipole rotation triggered by electromagnetic radiation (300 MHz–300 GHz) to selectively extract target compounds [36]. Microwaves generate heat through ion conduction and dipole rotation: ion movement creates resistance-based heat, while dipole molecules realign with the electric field, causing friction and collisions. This also enhances hydrogen bonding, improving solubility and extraction efficiency [41,42]. MAE outperformed Soxhlet extraction in both oil recovery and time efficiency when extracting oil from *Moringa oleifera* seeds utilizing ethanol. Specifically, MAE yielded 16% more oil and reduced the extraction duration from 8 h to merely 1 h, thereby illustrating its superior efficiency [43]. In another study, the extraction of the edible marine alga *Undaria pinnatifida* using an n-hexane–acetonitrile–methanol solvent system (5:5:3, *v*/*v*/*v*), combined with MAE and high-speed counter-current chromatography (HSCCC), yielded highly purified phytosterols, with fucosterol and 24-methylencholesterol achieving purities of 98.2% and 97.0%, respectively. The extraction yields were 1.21 mg/g for fucosterol and 0.75 mg/g for 24-methylencholesterol [44]. In a separate investigation, MAE was used to extract β-sitosterol and its glucoside from *Agave angustifolia* Haw bagasse under open-system conditions at 21–23 °C for 9 s, using ethanol as the extraction solvent. The analytical results revealed that the extract contained 103.6 mg/g of β-sitosterol and 61.6 mg/g of β-sitosterol glucoside. MAE enabled the rapid and efficient extraction for compounds under mild conditions, achieving high yields within an extremely short time [45]. In a more recent study, Soxhlet extraction and MAE were compared for oil extraction from *Tamarindus indica* seeds, with both methods employing petroleum ether as a non-polar solvent [46]. MAE yielded approximately 71% oil, significantly higher than the 50% yield obtained through Soxhlet extraction. Moreover, phytosterol compounds such as campesterol and cycloartal exhibited strong inhibitory activity against the LDL receptor protein, with binding energies of −12.00 and −12.53 kcal/mol, respectively, indicating their potential as effective LDL-lowering agents.

#### 2.2.3. Pressurized Liquid Extraction (PLE)

PLE is a rapid, eco-friendly extraction method using high temperature and pressure to efficiently isolate bioactives like polysaccharides and PUFAs within 5–20 min [47]. Additionally, it involves mixing the sample with an inert material, applying heat (75–200 °C) and pressure (up to 100 atm) in a steel vessel, and using compressed gas to transfer the extract. Compared to Soxhlet, PLE offers faster extraction, lower solvent use, and built-in filtration [34]. In one study, the use of PLE for extracting β-sitosterol from almonds in methanol resulted in a yield of 1.16 ± 0.15 mg/g, which was slightly higher than the yield obtained using saponification, reported as 1.15 ± 0.07 mg/g [48]. Another investigation applied PLE to rapeseed using petroleum ether at 100 °C and 10 MPa for 6 min (two cycles), analyzing eight different sterols. Among them, β-sitosterol was the most abundant, with a concentration of 4242.28 mg/kg, demonstrating PLE’s effectiveness in phytosterol extraction and profiling in oilseed crops [49].

Further comparison with Soxhlet extraction demonstrated PLE’s superior performance in extracting rice bran oil. The oil yield from PLE reached 27.22%, exceeding the 20.9% yield obtained via Soxhlet. Moreover, β-sitosterol content in the PLE-extracted oil was higher (3.91 mg/g) than that of Soxhlet (3.34 mg/g). PLE also resulted in significantly greater antioxidant activity (1.934 mmol TE/g) and total phenolic content. Importantly, while Soxhlet extraction typically employs petroleum-based solvents, PLE can utilize ethanol, which is classified as “generally recognized as safe” (GRAS), thereby offering a safer and more environmentally sustainable alternative [50].

#### 2.2.4. Solid-Phase Extraction (SPE)

SPE is a widely used technique for isolating, purifying, and concentrating target compounds from liquid samples by passing them through a cartridge containing a selective solid-phase material. SPE types include reversed-phase, normal-phase, ion-exchange, and mixed-mode, depending on the solid-phase chemistry [51,52,53,54]. A study illustrated the development of an innovative monolithic column designed for the concentration of β-sitosterol from plant oil. Compared to standard C18 sorbents, the monolithic column exhibited superior permeability and significantly lower back pressure, not exceeding 2 MPa. Recovery rates for spiked β-sitosterol ranged from 90.96% to 103.56%, underscoring the high efficiency and analytical reliability of the approach [55]. Another study employed a sample preparation method that combined saponification with SPE to enable precise quantification of phytosterols in edible vegetable oils [56]. Organic solvents such as hexane, diethyl ether, and acetone were used to achieve effective component separation. Notably, rice bran oil was found to contain a total of 13.46 g/kg of phytosterols, with 76.22% present in bound form. These findings demonstrate the capability of SPE-based techniques to separate and quantify both free and bound phytosterols, even within the complex lipid matrices of various vegetable oils.

#### 2.2.5. Solid-Phase Microextraction (SPME)

SPME is a solvent-saving technique used in gas chromatography–mass spectrometry (GC-MS) and liquid chromatography–mass spectrometry (LC-MS) that extracts analytes from gas, liquid, or solid samples via adsorption onto a coated fiber, followed by thermal or solvent desorption for analysis [57]. The effectiveness of SPME relies on its fiber coatings—such as polydimethylsiloxane (PDMS), carboxen/polydimethylsiloxane (CAR/PDMS), and divinylbenzene (DVB)—which are specifically engineered to selectively extract compounds based on their polarity and volatility [58]. In one study, a novel molecularly imprinted polymer (MIP)-based SPME method was employed to extract β-sitosterol from egg yolk, milk, and olive oil in acetonitrile. This method demonstrated excellent reusability across at least 20 cycles, with recovery rates for β-sitosterol ranging from 92% to 101%, demonstrating its reliability and efficiency [59]. Another study developed a simplified and rapid method for the analysis of phytosterols and cholesterol precursors in human serum by coupling SPME with GC-FID [60]. Serum samples were first saponified using KOH in ethanol, and polyacrylate fibers preconditioned with bis(trimethylsilyl)trifluoroacetamide (BSTFA) vapor were used for simultaneous extraction and on-fiber derivatization at 90 °C for 90 min. Key analytes included sitosterol, sitostanol, and desmosterol, with measured sitosterol concentrations ranging from 1.24 to 11.56 µg/g and sitostanol levels reaching up to 16.04 µg/g. This approach enabled high selectivity and sensitivity using low sample volumes and no solvent, making it particularly suitable for clinical applications. SPME offers several advantages, including reduced solvent consumption, simplified sample preparation, high sensitivity, and compatibility with automation. However, limitations such as limited fiber lifespan, sensitivity to harsh extraction conditions, and variability in recovery efficiency remain important considerations for its broader application.

#### 2.2.6. Liquid-Phase Extraction

Conventional LLE uses solubility differences between immiscible solvents but is limited by high solvent use, labor intensity, and moderate efficiency [61]. Liquid–liquid microextraction (LLME) improves upon traditional LLE by using less solvent, offering faster extraction, lower cost, and higher enrichment, though poor dispersion can limit analyte recovery [62].

To address these limitations, DLLME was developed, where a mixture of extraction and disperser solvents is rapidly injected into an aqueous sample, forming a turbid solution with fine droplets that enhance analyte transfer [63,64]. DLLME provides rapid, user-friendly extraction with low solvent use and high enrichment, making it ideal for analytical use. Recent improvements include greener solvents, better dispersion, and integration with other methods, expanding its applications in environmental, food, and clinical fields [65]. A recent study developed a simple and efficient DLLME method for the extraction of four phytosterols—β-sitosterol, stigmasterol, brassicasterol, and campesterol—from milk. Compared to conventional organic solvent-based DLLME, the deep eutectic solvent (DES)-based DLLME method demonstrated significantly improved analytical performance, with approximately fourfold lower limits of detection (LOD) and fivefold lower limits of quantification (LOQ) for all four phytosterols. In addition, it achieved high extraction recoveries ranging from 82% to 91%. This method was found to be rapid, sensitive, and effective, making it highly suitable for the extraction of phytosterols from milk [66]. Another advanced method combined dual ultrasound-assisted dispersive liquid–liquid microextraction (dual-UADLLME), microwave-assisted derivatization (MAD), and UHPLC-MS/MS for the quantitative analysis of phytosterols. The optimized extraction procedure used minimal solvent volumes—bromocyclohexane (70 µL), ethanol (250 µL), bromobenzene (70 µL), and acetonitrile (220 µL)—and achieved rapid extraction within 2 min. Dual-UADLLME significantly reduced matrix effects compared to conventional DLLME (from 38.7–88.9% to 88.3–108.5%) and markedly improved enrichment factors, achieving values of 207 for campesterol, 176 for β-sitosterol, and 193 for stigmasterol. The method demonstrated excellent sensitivity, with LODs ranging from 0.005 to 0.015 ng/mL and LOQs from 0.030 to 0.10 ng/mL. When applied to various edible oils such as sunflower oil, olive oil, and corn oil, β-sitosterol was found as the most abundant phytosterol, with concentrations ranging from 2745 to 5140 µg/g [67].

### 2.3. Phytosterol Analysis Techniques

Phytosterols share a structural similarity with other sterols, such as cholesterol, making their differentiation analytically challenging. Due to their common tetracyclic ring structure and C-17 side chain, accurate identification and quantification in complex matrices require advanced separation techniques like HPLC, GC, or SFC, often coupled with MS or FIDs. These methods provide the sensitivity and resolution needed for precise phytosterol analysis.

#### 2.3.1. Liquid Chromatography

HPLC is a widely utilized technique for the analysis of phytosterols. The columns commonly used for the detection of phytosterols include C8, phenyl, and C18 columns, which effectively facilitate the separation of these compounds [68,69,70]. Recent studies favor C18 over C8 columns for phytosterol separation from complex mixtures due to its longer alkyl chain, which enhances hydrophobic interactions and improves analyte retention and separation.

Various analytical techniques for phytosterol quantification differ in terms of sensitivity, analysis time, and the associated advantages and limitations. The HPLC-DAD method is widely employed due to its simple instrumentation and short analysis time (approximately 2–6 min), though it offers limited sensitivity and selectivity [71]. More recently, fast chromatography–MS/MS methods have been developed, offering a rapid analysis time of approximately 1.3 min and minimal sample preparation [72]; however, potential interference among analytes remains a concern. Therefore, the optimal analytical technique should be selected based on the specific analytical objectives, sample complexity, and budgetary constraints.

In one recent study, LC-MS/MS was employed to quantify five minor phytosterols—lophenol, 24-methyl-lophenol, 24-ethyl-lophenol, cycloartanol, and 24-methylene-cycloartanol—extracted from *Aloe vera* gel using a chloroform/methanol (2:1, *v/v*) solvent system. This method allowed direct analysis derivatization or saponification. LOQ ranged from 2.3 to 4.1 ng/mL, with recovery rates between 95% and 105%, indicating high accuracy. Intra- and inter-day precisions ranged from 2.6 to 6.4% and from 3.8 to 7.3%, respectively, confirming robust reproducibility. Phytosterol content in *Aloe vera* gel powder (AVGP) was determined to be 9.6–33.2 μg/g, supporting the method’s reliability for quantifying trace levels in functional food ingredients and long-term storage products [73].

Another study applied APCI-LC-MS/MS for the analysis of phytosterols in edible oils, eliminating the need for derivatization. Oil samples were first saponified using ethanolic KOH, extracted with hexane, and analyzed using an isocratic mobile phase of acetonitrile/methanol (99:1, *v/v*). Six phytosterols—brassicasterol, campesterol, cycloartenol, β-sitosterol, stigmasterol, and lupeol—were quantified within a 4 min run time. Among the tested samples, corn oil had the highest amount of β-sitosterol (4.35 mg/g), while canola oil showed the highest amount of campesterol (1.84 mg/g) and brassicasterol (488 µg/g). The method demonstrated excellent sensitivity, with LODs ranging from 2 to 25 ng/mL and LLOQs from 10 to 100 ng/mL. These performance metrics allowed for quantification down to 1.0 µg/g for campesterol and lupeol. Compared to conventional GC-MS and HPLC-UV, this approach offered faster analysis and superior sensitivity for phytosterol profiling in complex oil matrices [74].

#### 2.3.2. Gas Chromatography (GC)

GC is a powerful technique for the separation and quantification of volatile, thermally stable compounds. It operates using a column coated with a stationary phase and a gaseous mobile phase to separate analytes based on their physicochemical interactions. GC provides high-resolution and rapid analysis, and it is compatible with various detectors such as flame ionization detectors (FIDs), commonly used for sterol analysis, and mass spectrometry (GC-MS), which offers structural identification capabilities. However, analytes must be volatile and thermally stable to be suitable for GC analysis. This limits the direct applicability of GC to certain compounds, including many bioactive lipids and polar molecules.

Phytosterols, while thermally stable, exhibit low volatility and high polarity, making them unsuitable for direct GC analysis. To address this limitation, derivatization is commonly employed to improve their volatility and chromatographic performance. Frequently used derivatization reagents include N-Methyl-N-(trimethylsilyl)trifluoroacetamide (MSTFA) and bis(trimethylsilyl)trifluoroacetamide (BSTFA), often used in conjunction with 1% trimethylchlorosilane (TMCS) [75]. A particularly effective derivatization method utilizes a mixture of MSTFA, 1,4-dithioerythritol (DTE), and trimethyliodosilane (TMSI) in a ratio of 5 mL:10 mg:10 μL. This combination converts phytosterols into their trimethylsilyl (TMS) derivatives, significantly enhancing their volatility, peak resolution, and quantification via GC. This approach has proven to be a simple, rapid, and sensitive method for analyzing phytosterols such as β-sitosterol and campesterol [76].

Detection limits vary by instrumentation and protocol. Using GC-FID, detection limits for phytosterols typically range from 0.02 to 0.2 mg/kg depending on the derivatization method and matrix complexity. In contrast, GC-MS conducted in selected ion monitoring (SIM) mode can achieve much lower detection limits, ranging from 5 to 50 ng/mL [77,78].

Resolution and overall method performance in GC analysis are influenced by several factors, including column type, temperature programming, and sample matrix. Complex matrices such as oils or dairy products require appropriate clean-up procedures prior to derivatization [79]. Therefore, careful selection of derivatization reagents, detector type, and sample preparation strategy is critical for achieving reliable and sensitive phytosterol analysis using GC.

Notably, a derivatization-free GC-MS/MS method has also been developed for the rapid analysis of phytosterols and cholesterol in food matrices [80]. This approach utilizes multiple reaction monitoring (MRM) mode to achieve high specificity and accuracy, enabling analysis to be completed within 20 min. While this method significantly enhances analytical efficiency and practicality, it exhibits a relatively higher limit of quantification (2 mg/kg) compared to derivatized GC methods. Recovery rates range from 91% to 95%, and precision spans 2–12%, demonstrating moderate variability depending on the matrix and analyte.

#### 2.3.3. Supercritical Fluid Chromatography (SFC)

SFC is a chromatographic technique that employs supercritical fluid as the mobile phase, thereby combining characteristics of both GC and LC. Carbon dioxide is the most commonly utilized supercritical fluid due to its favorable critical temperature and pressure. The polarity of carbon dioxide can be modified by the incorporation of co-solvents (modifiers) such as methanol, ethanol, or isopropanol, which enhances the solubility of polar compounds [81,82]. SFC provides high diffusion rates, enabling quick analysis similar to GC, while also decreasing the use of organic solvents, which makes it a more eco-friendly option. Additionally, SFC can successfully analyze semi-volatile compounds, and its lower viscosity compared to LC reduces diffusion in the column, resulting in better separation efficiency and enhanced resolution [82,83]. A novel analytical technique utilizing supercritical fluid chromatography coupled with atmospheric pressure chemical ionization tandem mass spectrometry (SFC-APCI-MS/MS) has been established to concurrently profile seven phytosterols—namely, brassicasterol, campesterol, stigmasterol, β-sitosterol, δ-5-acenasterol, cycloartenol, and lupeol—present in coconut oil and palm oil. This method effectively identified and quantified the phytosterols in both types of oil. The LOD for the individual phytosterols ranged from 1 to 15 ng/mL, while the LOQ varied from 5 to 40 ng/mL [84]. Another study utilized SFC coupled with quadrupole time-of-flight mass spectrometry (SFC-QTOF-MS) for the rapid and sensitive analysis of squalene, tocopherols, and phytosterols in walnut oil, without the need for sample derivatization or extensive preparation. Compared to conventional LC and GC methods, this approach offered a shorter analysis time and higher sensitivity (LOD: 0.05–0.20 ng/mL), along with excellent quantification accuracy, with recovery rates ranging from 70.61% to 101.44%. Moreover, β-sitosterol concentrations reached up to 1385.18 mg/kg. These results underscore the utility of SFC-QTOF-MS as an effective platform for profiling liposoluble micronutrients across diverse walnut cultivars and processing conditions [85].

**Table 1:** Relevant literature on phytosterol extraction and analysis techniques.

Sample Matrix	Analyzed Phytosterols	Extraction Methods	Determination Methods	Extract Yield (mg/g)	References
Linseed oil	β-Sitosterol	Saponification, LLE with diethyl ether	RPLC-SALDI MS	2.680	[86]
	Stigmasterol			0.850	
	Campesterol			1.670	
	Brassicasterol			0.680	
Niger seed oil	β-Sitosterol	Saponification, LLE with diethyl ether, SPE	TMS-derivatized samples were analyzed by GC-MS	2.779	[29]
	Stigmasterol			0.920,	
	Campesterol			0.870	
	Cycloartenol			0.131	
*P. lactiflora* Pall seed oils	Campesterol	Maceration with n-hexane, UAE	HPLC	0.37345	[87]
	Stigmasterol			0.04596	
	β-Sitosterol			2.47376	
	Isofucosterol			0.2347	
	Δ^7^-Avenasterol			0.10951	
*P. lactiflora* Pall seed oils	Campesterol	SFE	HPLC	0.31535	[87]
	Stigmasterol			0.02383	
	β-Sitosterol			3.28167	
	Isofucosterol			0.37213	
	Δ^7^-Avenasterol			0.17138	
*P. lactiflora* Pall seed oils	Campesterol	Pressing	HPLC	0.32799	[87]
	Stigmasterol			0.04674	
	β-Sitosterol			2.7845	
	Isofucosterol			0.30375	
	Δ^7^-Avenasterol			0.14032	
Olive drupes	Brassicasterol	UAE, saponification, LLE with hexane, SPE	TMS-derivatized samples were analyzed by GC-MS	0.004	[88]
	Campesterol			0.0068	
	Stigmasterol			0.0198	
	β-Sitosterol			2.3997	
Canola oil (Richfood)	β-Sitosterol	Saponification, LLE with n-hexane	APCI-LC-MS/MS	3.597	[74]
	Campesterol			1.837	
	Brassicasterol			0.4879	
	Stigmasterol			0.034	
	Cycloartenol			0.087	
	Lupeol			<LLOQ	
Olive oil (Richfood)	β-Sitosterol	Saponification, LLE with n-hexane	APCI-LC-MS/MS	1.209	[74]
	Campesterol			0.0542	
	Brassicasterol			<LLOQ	
	Stigmasterol			0.0236	
	Cycloartenol			0.338	
	Lupeol			0.0196	
Walnut oil	Campesterol	In situ direct analysis	SFC-QTOF-MS	0.03181	[85]
	β-Sitosterol			0.86884	
	Stigmasterol			0.04065	
Hemp oil (Living Harvest)	β-Sitosterol	Saponification, LLE with n-hexane	APCI-LC-MS/MS	3.16	[74]
	Campesterol			0.706	
	Brassicasterol			<LLOQ	
	Stigmasterol			0.1136	
	Cycloartenol			0.169	
	Lupeol			<LLOQ	
Almond oil (International Collection)	β-Sitosterol	Saponification, LLE with n-hexane	APCI-LC-MS/MS	0.940	[74]
	Campesterol			0.1842	
	Brassicasterol			0.0146	
	Stigmasterol			0.1569	
	Cycloartenol			0.139	
	Lupeol			0.0195	
Hazelnut oil (International Collection)	β-Sitosterol	Saponification, LLE with n-hexane	APCI-LC-MS/MS	0.472	[74]
	Campesterol			0.301	
	Brassicasterol			0.0015	
	Stigmasterol			0.0097	
	Cycloartenol			0.0054	
	Lupeol			0.0035	
Walnut oil (International Collection)	β-Sitosterol	Saponification, LLE with n-hexane	APCI-LC-MS/MS	1.222	[74]
	Campesterol			0.0899	
	Brassicasterol			0.0026	
	Stigmasterol			0.00131	
	Cycloartenol			0.244	
	Lupeol			<LLOQ	
Almonds	β-Sitosterol	PLE with methanol	LC-DAD	1.16 ± 15	[48]
Grape seed oil (International Collection)	β-Sitosterol	Saponification, LLE with n-hexane	APCI-LC-MS/MS	1.913	[74]
	Campesterol			0.3935	
	Brassicasterol			<LLOQ	
	Stigmasterol			0.340	
	Cycloartenol			0.2617	
	Lupeol			0.0285	
Sesame oil (Spectrum)	β-Sitosterol	Saponification, LLE with n-hexane	APCI-LC-MS/MS	3.215	[74]
	Campesterol			0.880	
	Brassicasterol			<LLOQ	
	Stigmasterol			0.418	
	Cycloartenol			0.1564	
	Lupeol			0.0128	
Avocado oil (Spectrum)	β-Sitosterol	Saponification, LLE with n-hexane	APCI-LC-MS/MS	2.629	[74]
	Campesterol			0.4043	
	Brassicasterol			<LLOQ	
	Stigmasterol			0.1726	
	Cycloartenol			0.3471	
	Lupeol			0.0181	
Sunflower oil (Richfood)	β-Sitosterol	Saponification, LLE with n-hexane	APCI-LC-MS/MS	2.178	[74]
	Campesterol			0.3178	
	Brassicasterol			0.0135	
	Stigmasterol			0.301	
	Cycloartenol			0.1745	
	Lupeol			0.0302	
Peanut oil (Richfood)	β-Sitosterol	Saponification, LLE with n-hexane	APCI-LC-MS/MS	1.030	[74]
	Campesterol			0.278	
	Brassicasterol			0.019	
	Stigmasterol			0.1541	
	Cycloartenol			0.0582	
	Lupeol			0.0128	
Corn oil (Richfood)	β-Sitosterol	Saponification, LLE with n-hexane	APCI-LC-MS/MS	4.354	[74]
	Campesterol			1.555	
	Brassicasterol			<LLOQ	
	Stigmasterol			0.703	
	Cycloartenol			0.2731	
	Lupeol			<LLOQ	
Macadamia nut oil (Olivado)	β-Sitosterol	Saponification, LLE with n-hexane	APCI-LC-MS/MS	1.915	[74]
	Campesterol			0.1378	
	Brassicasterol			<LLOQ	
	Stigmasterol			0.0071	
	Cycloartenol			0.0085	
	Lupeol			<LLOQ	
Sunflower oil (Hollywood)	β-Sitosterol	Saponification, LLE with n-hexane	APCI-LC-MS/MS	1.349	[74]
	Campesterol			0.369	
	Brassicasterol			0.0111	
	Stigmasterol			0.1594	
	Cycloartenol			0.14	
	Lupeol			0.042	
*U. pinnatifida*	Fucosterol	MAE, saponification, LLE with n-hexane	HPLC-UV and GC-MS	1.21	[44]
	24-Methylenecholesterol			0.16	
Coconut oil	Brassicasterol	Solvent extraction with heptane, saponification	SFC-APCI-MS/MS	9.41 ± 0.39	[84]
	Campesterol			4.34 ± 0.33	
	δ-5-Acenasterol			20.94 ± 0.60	
	Stigmasterol			21.51 ± 0.46	
	β-Sitosterol			32.71 ± 2.75	
	Lupeol			18.89 ± 0.89	
	Cycloartenol			18.97 ± 1.26	
Palm oil	Brassicasterol	Solvent extraction with heptane, saponification	SFC-APCI-MS/MS	4.93 ± 0.76	[84]
	Campesterol			4.14 ± 0.12	
	δ-5-Acenasterol			12.70 ± 0.82	
	Stigmasterol			13.20 ± 0.92	
	β-Sitosterol			31.97 ± 0.28	
	Lupeol			16.30 ± 0.45	
	Cycloartenol			18.49 ± 0.26	

LLE, liquid–liquid extraction; RPLC-SALDI MS, reversed-phase liquid chromatography with surface-assisted laser desorption/ionization mass spectrometry; SPE, solid-phase extraction; TMS, trimethylsilyl; GC-MS, gas chromatography–mass spectrometry; UAE, ultrasonic-assisted extraction; HPLC, high-performance liquid chromatography; LC-MS/MS, liquid chromatography–tandem mass spectrometry; SFC-QTOF-MS, supercritical fluid chromatography coupled with quadrupole time-of-flight mass spectrometry; PLE, pressurized liquid extraction; SFC-APCI-MS/MS, supercritical fluid chromatography–atmospheric pressure chemical ionization–tandem mass spectrometry; SFE, supercritical fluid extraction; LLOQ, limit of quantitation.

## 3. Health Benefits of Phytosterols

Phytosterols are plant-derived sterols characterized by a fundamental four-ring steroid structure that bears resemblance to cholesterol; however, they are distinguished primarily by variations in their side-chain composition and the presence of additional double bonds. Due to this structural similarity, phytosterols engage in competition with cholesterol within the gastrointestinal tract, thereby playing a significant role in the regulation of cholesterol levels. This section provides a comprehensive examination of the biological functions and mechanisms by which phytosterols exert their effects, supported by recent empirical evidence (Table 2).

### 3.1. Cardiovascular Diseases (CVDs) and Phytosterols

CVDs continue to be the primary cause of illness and death globally, primarily influenced by risk factors that can be changed, including high cholesterol, high blood pressure, diabetes, and obesity. High levels of LDL-C are closely linked to the onset of atherosclerosis, which is the fundamental condition behind numerous CVDs [104]. Therefore, both dietary changes and medication strategies focused on lowering LDL-C are essential elements in managing cardiovascular risk.

Phytosterols, which are plant-based sterols and stanols that resemble cholesterol, have garnered considerable interest among the numerous dietary compounds researched for their ability to lower lipids. Clinical studies and meta-analyses have repeatedly shown that consistent consumption of phytosterols can lead to a reduction in LDL-C levels by 5–15%, influenced by the amount taken and the initial cholesterol levels [91,105,106]. This phenomenon is predominantly facilitated by the suppression of intestinal cholesterol absorption. A comprehensive understanding of the molecular mechanisms that regulate phytosterol metabolism is crucial for a complete appreciation of their contribution to the prevention of cardiovascular diseases and the development of dietary intervention strategies.

**Figure 2:**
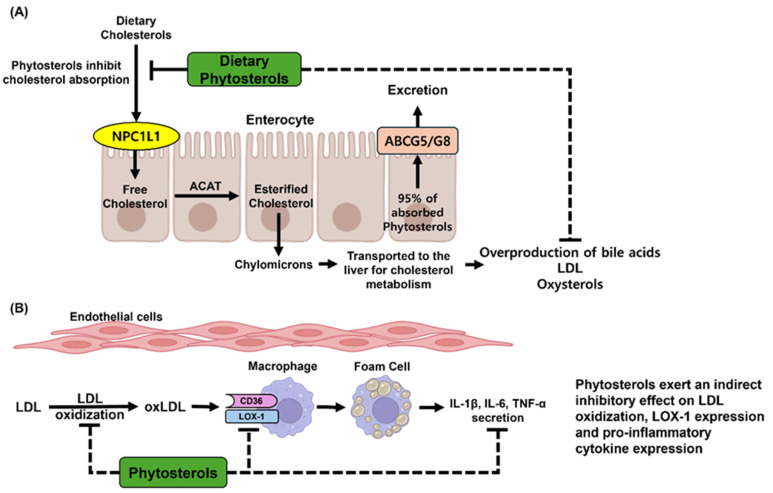
Physiological effects of phytosterols in the human body. (**A**) Dietary phytosterols are competitively absorbed with cholesterol via the NPC1L1 transporter in the intestinal epithelium. Phytosterols inhibit intestinal cholesterol absorption, thereby suppressing downstream cholesterol metabolism. Approximately 95% of the absorbed phytosterols are effluxed back into the intestinal lumen via the ABCG5/G8 transporter. As a result, phytosterols help to reduce the overproduction of bile acids, LDL, and oxidized cholesterol derivatives (oxysterols), all of which are associated with chronic metabolic diseases. (**B**) Phytosterols suppress the synthesis of LDL, which can accumulate in vascular endothelial cells. By reducing LDL deposition, phytosterols help to prevent the formation of oxLDL within the endothelium. Consequently, this inhibits the transformation of macrophages into foam cells. Fundamentally, phytosterols attenuate the expression of pro-inflammatory cytokines in foam cells, thereby contributing to the prevention of vascular inflammation and the development of atherosclerosis.

Upon consumption, phytosterols engage with Niemann-Pick C1-like 1 (NPC1L1), a crucial sterol transporter that facilitates cholesterol uptake. While NPC1L1 primarily mediates the endocytosis of cholesterol into enterocytes, phytosterols serve as competitive substrates, thereby diminishing the efficiency of cholesterol absorption [10]. Following their internalization, both cholesterol and phytosterols undergo intracellular trafficking, during which they are directed for efflux by ATP-binding cassette transporter G5/G8 (ABCG5/G8). This active transport mechanism preferentially expels phytosterols back into the intestinal lumen, resulting in a significantly lower absorption rate (less than 5%) for phytosterols compared to cholesterol [107]. Consequently, the majority of dietary phytosterols are excreted via feces, while a small proportion that is absorbed enters systemic circulation through chylomicron-mediated transport, ultimately reaching the liver, where hepatic ABCG5/G8 further promotes their biliary excretion (Figure 2A).

The distinctive metabolic properties of phytosterols play a crucial role in the regulation of cholesterol homeostasis within the body, specifically aiding in the reduction in LDL-C. LDL-C is recognized as a significant risk factor for CVDs, and elevated levels of LDL-C can result in its accumulation and oxidation in the arterial wall, thereby facilitating the development of foam cells. This mechanism accelerates the progression of atherosclerosis, consequently heightening the risk of cardiovascular events.

Evidence supporting these effects is derived from a study utilizing a Syrian hamster model of dyslipidemia, which was induced by a high-fat diet over a duration of eight weeks. In this investigation, the high-fat diet was supplemented with *Cajanus cajan* L. (pigeon pea) oil, which contained 191.6 mg of β-sitosterol, 292.8 mg of campesterol, 292.8 mg of stigmasterol, and a total of 1547.5 mg of phytosterols per 100 g. The results indicated a significant reduction in serum levels of LDL, TC, and triglyceride (TG). Additionally, the treatment led to an increase in the fecal excretion of TG and bile acids. Moreover, *C. cajan* oil was observed to upregulate the hepatic expression of CPT-1, thereby enhancing β-oxidation and mitigating fatty acid accumulation. It also elevated the expression of CYP7A1 and LDL receptors, facilitating the conversion of cholesterol into bile acids and promoting cholesterol excretion [89]. In an additional investigation, male C57BL/6 mice subjected to a 4-week high-fat diet, which induced fat accumulation, were administered an oral dose of 100 mg/kg of a phytosterol preparation exhibiting 95% purity. This preparation comprised more than 40% β-sitosterol, 20% campesterol, and over 14% stigmasterol. The administration of this treatment led to a notable decrease in serum TG, TC, and LDL-C levels, while HDL-C levels were significantly elevated [90]. A clinical trial has demonstrated that a daily consumption of 1.5–2.15 g of phytosterols can lead to a reduction in cholesterol absorption by 30–40% and a decrease in LDL-C levels by approximately 8.9%, which may contribute to a lower risk of CVDs [91,92]. Furthermore, the use of phytosterols in conjunction with statins has been found to produce an additive effect in lowering LDL-C levels [108], indicating a potential enhancement in cardiovascular protection.

### 3.2. Anti-Inflammatory Effects of Phytosterols

Chronic inflammation is a pivotal factor in the onset and advancement of various diseases, including cardiovascular conditions, metabolic disorders, neurodegenerative diseases, and inflammatory bowel diseases. Extended or dysregulated inflammatory responses can result in tissue damage, immune dysfunction, and the aggravation of pathological states. Recently, phytosterols have garnered significant attention, not only for their well-documented cholesterol-lowering properties but also for their emerging role in modulating anti-inflammatory signaling pathways, positioning them as promising candidates for the prevention and management of diseases associated with inflammation.

Phytosterols play a crucial role in promoting cardiovascular health by reducing circulating levels of LDL-C, the principal cholesterol carrier in the bloodstream. LDL-C interacts with endothelial cells through apolipoprotein B-100 (ApoB-100)-mediated translocation and can accumulate within the subendothelial space of blood vessels [68]. In this microenvironment, LDL-C is particularly vulnerable to oxidation by ROS and enzymatic catalysts such as myeloperoxidase (MPO). This oxidative modification results in the formation of oxidized LDL (oxLDL), a highly atherogenic variant of LDL that significantly contributes to the initiation and progression of atherosclerosis. OxLDL is recognized and internalized by macrophages via scavenger receptors (CD36 and LOX-1). Unlike native LDL, oxLDL is not regulated by the intracellular cholesterol homeostasis feedback mechanism, leading to excessive lipid accumulation and the transformation of macrophages into foam cells [109]. Foam cells are instrumental in the development of fatty streaks, which represent the initial stage of atherosclerotic lesions. As these fatty streaks persist and expand, chronic inflammatory responses are activated, characterized by the recruitment of additional immune cells, increased secretion of pro-inflammatory cytokines (e.g., IL-1β, IL-6, and TNF-α) (Figure 2B) [110,111], and further oxidation of lipoproteins. Over time, these processes culminate in the formation of atherosclerotic plaques, which consist of a lipid-rich necrotic core encased by a fibrous cap. The stability of these plaques is a critical factor in determining cardiovascular risk; unstable plaques are susceptible to rupture, potentially leading to the formation of acute thrombi that can precipitate severe cardiovascular events such as myocardial infarction and ischemic stroke. Consequently, by lowering LDL-C levels, phytosterols provide protective effects not only by attenuating the progression of atherosclerosis but also by alleviating the associated inflammatory responses.

In a particular investigation, pretreatment of RAW 264.7 macrophages with β-sitosterol at a concentration of 10 μM for a duration of 3 h, in conjunction with stimulation by oxidized low-density lipoprotein (oxLDL) at a concentration of 40 μg protein/mL to simulate foam cell formation, resulted in significant inhibition of the oxLDL-induced production of ROS, including H_2_O_2_. This attenuation of ROS production was associated with a decrease in the release of arachidonic acid, a reduction in the expression of cyclooxygenase-2 (COX-2), and a lower synthesis of PGE2. Furthermore, the inhibitory effects were markedly enhanced when β-sitosterol was administered concurrently with tyrosol, a polyphenolic compound, demonstrating statistically significant improvements [93].

Additional research utilizing RAW 264.7 macrophages stimulated with LPS (1 μg/mL) as an inflammatory model has shown that phytosterols, including ergosterol acetate, ergosterol, β-sitosterol, campesterol, and stigmasterol, exert anti-inflammatory effects by downregulating critical inflammatory mediators such as COX-2, inducible nitric oxide synthase (iNOS), and extracellular signal-regulated kinase (ERK) [94]. These molecules serve as essential constituents of the nuclear factor-kappa B (NF-κB) and mitogen-activated protein kinase (MAPK) signaling pathways, both of which play a pivotal role in the regulation of inflammatory gene expression and the activation of immune cells.

In vivo investigations further substantiate the anti-inflammatory properties of phytosterols. For example, in a model of colitis induced by DSS, the administration of a 4RF21 diet, delivering phytosterols at a dosage of 400 mg/kg/day, has been observed to mitigate the progression of IBD. Treatment with phytosterols resulted in a reduction in histopathological damage within intestinal tissues and a decrease in the expression of pro-inflammatory cytokines in the colonic mucosa [95]. These results indicate that phytosterols possess biological activities that extend beyond their lipid-lowering effects, potentially influencing immune responses and alleviating inflammation in conditions beyond cardiovascular health.

### 3.3. Anticancer Effects of Phytosterols

Dietary habits, lifestyle choices, and stress are significant contributors to the etiology of cancer. Nevertheless, the role of phytosterols in cancer research, particularly their potential anticancer effects, remains relatively underinvestigated. Phytosterols act as inhibitors of HDACs, which are involved in the epigenetic regulation of gene expression associated with cancer progression. Notably, β-sitosterol (80 μM) and lupeol (40 μM), both classified as phytosterols, have demonstrated the ability to downregulate the expression of HDAC1 and HDAC2 in prostate cancer cell lines PC3 and DU145 [96].

One study reported that, stigmasterol, isolated from Azadirachta indica, was assessed for its chemopreventive potential in a DMBA-induced skin carcinoma model in Swiss albino mice. A single topical dose of DMBA (100 μg/100 μL acetone) followed by 1% croton oil induced skin tumors, while stigmasterol (400 mg/kg) was administered orally three times weekly for 16 weeks. Stigmasterol significantly reduced tumor incidence and multiplicity, inhibited lipid peroxidation and ROS, and enhanced antioxidant enzymes including GSH, superoxide dismutase, and catalase. It also improved histological features of the skin and protected against DMBA-induced DNA damage. These effects suggest that stigmasterol exerts anticancer activity through antioxidants and antigenotoxic mechanisms [97].

Findings from a clinical trial indicate that, among the 1802 cancer patients studied, those with a high dietary intake of phytosterols demonstrated a significantly reduced risk of developing colon cancer in comparison to individuals with lower intake levels. Specifically, participants in the highest quartile of phytosterol consumption experienced a 50% reduction in the risk of colon cancer when compared to those in the lowest quartile [98].

### 3.4. Antidiabetic Effects of Phytosterols

Diabetes mellitus is a chronic metabolic disorder characterized by elevated blood glucose levels, which arise from deficiencies in insulin secretion, insulin action, or a combination of both. The persistent hyperglycemia associated with diabetes can lead to severe complications, including cardiovascular disease, neuropathy, nephropathy, and retinopathy. Type 2 diabetes, the most common disease, is closely associated with obesity, unhealthy dietary practices, and sedentary lifestyles. As the global prevalence of diabetes continues to escalate, there is a growing interest in identifying safe and effective dietary interventions that can aid in the regulation of glucose metabolism and enhance insulin sensitivity. Among these interventions, phytosterols have emerged as promising bioactive compounds with potential antidiabetic effects.

In a particular study, treatment with stigmasterol (50 μg/mL) in INS-1 insulinoma cells and human islets subjected to high-glucose and -palmitate (HGP) conditions (30 mM glucose, 0.5 mM palmitate) effectively mitigated the production of ROS induced by glucolipotoxicity. This intervention resulted in the normalization of intracellular cholesterol levels and the upregulation of sterol regulatory element-binding transcription factor 2 (Srebf2), a transcription factor that regulates cholesterol synthesis and uptake, as well as LDL receptors, the receptors responsible for cholesterol endocytosis. Furthermore, stigmasterol modulates F-actin to facilitate insulin exocytosis and operates through a mechanism distinct from that of macrophages, without increasing ATP-binding cassette transporter A1 (ABCA1) levels. Consequently, β-cell function was enhanced, and apoptosis was significantly reduced [99].

Furthermore, in a type 2 diabetes model established in albino rats through a 60-day regimen of a high-fat diet comprising 3% cholesterol, 1% cholic acid, and 30% coconut oil, in conjunction with drinking water enriched with 66% and 30% sucrose, oral administration of β-sitosterol (20 mg/kg) over a period of 30 days significantly reinstated the protein levels of the insulin receptor and GLUT4, which had diminished as a result of the high-fat and high-sucrose consumption [100]. A clinical study involving 276 patients diagnosed with GDM revealed that the intake of phytosterol-enriched spreads beginning in the third trimester led to significant improvements in maternal blood lipid profiles. Specifically, there were notable reductions in TAG, TC, LDL, and HOMA-IR levels, alongside increases in HDL and the QUICKI. Additionally, fasting plasma glucose (FPG) levels decreased by 9.5 mg/dL in patients consuming phytosterols compared to the general population, while insulin levels showed a reduction of 6.3 μIU/mL [101].

### 3.5. Neuroprotective Effects of Phytosterols

For pharmacological agents to have a positive impact on brain health, they must be able to effectively cross the BBB. The BBB is a carefully controlled endothelial structure that selectively permits the passage of molecules depending on their lipophilicity, size, and ability to engage with particular transport systems or receptors. As a result, only substances that are very lipophilic, have a low molecular weight, or are able to use specific transport methods can successfully penetrate this barrier [112]. Phytosterols are a group of naturally occurring sterols found in plants that have lipophilic characteristics, enabling them to cross the BBB. This ability allows them to influence neuronal growth, maintain cholesterol balance, and regulate neuroinflammation. Their function in preserving lipid balance in the brain is especially important, as imbalances in cholesterol metabolism have been linked to neurodegenerative diseases [19]. An excessive buildup of cholesterol in neurons negatively impacts brain function by changing the makeup and behavior of lipid rafts, which are specialized microdomains in the plasma membrane. These lipid rafts act as sites for the proteolytic processing of amyloid precursor protein (APP), promoting the BACE1 cleavage pathway and leading to increased production of Aβ. The accumulation of Aβ is a key feature of AD and other neurodegenerative disorders [113]. Research using animal models has shown that mice with high-cholesterol diets experience accelerated neurodegeneration [114].

From a mechanistic standpoint, phytosterols provide neuroprotective benefits by competitively blocking cholesterol absorption in the intestines, which leads to lower cholesterol levels in both the body and the brain. These compounds can penetrate the blood–brain barrier and help maintain cholesterol balance in neurons, reduce neuroinflammation, and alleviate synaptic dysfunction caused by cholesterol. By decreasing cholesterol buildup in neuronal membranes, phytosterols lower the activity of BACE1, which in turn reduces the production and aggregation of Aβ [19]. Therefore, while high cholesterol levels are known to be a risk factor for neurodegenerative diseases, phytosterols show promise as potential therapeutic agents that can combat cholesterol-related neurotoxicity. Their ability to influence lipid metabolism, modulate neuro-inflammatory processes, and reduce amyloidogenic activity makes them strong candidates for neuroprotective treatment strategies.

In this research, fucosterol isolated from *Eisenia stolonifera* was administered to the dorsal hippocampus of older rats at a dosage of 10 μmol/h for a duration of four weeks. To create cognitive impairment, Aβ_1–42_ was injected intraperitoneally at a dose of 25 mg/kg for seven consecutive days. After the treatment, the Morris water maze test was performed to evaluate cognitive function. The group treated with fucosterol showed a notable decrease in the time taken to reach the hidden platform, suggesting enhanced spatial learning and memory. Furthermore, the administration of fucosterol increased the expression of mature BDNF in the hippocampus [102].

A separate study showed that, in a model of cognitive impairment induced by AlCl_3_ in C57BL/6 mice, β-sitosterol (25 mg/kg) was given alongside AlCl_3_ (10 mg/kg) for a duration of 21 days. β-sitosterol was found to cross the BBB, lower levels of Aβ and AChE, improve memory performance, and boost brain GSH levels. It may also aid in the clearance of Aβ by inhibiting BACE1 or activating secretase enzymes. These results underscore the neuroprotective, anticholinesterase, and anti-inflammatory properties of β-sitosterol in the treatment of AD, although additional research is necessary to better understand its mechanisms [103].

## 4. Bioavailability of Phytosterols

Phytosterols cannot be produced by the human body, so they must be acquired through the diet or supplements. In healthy individuals, the absorption rate of phytosterols is typically below 5%, which is much lower than the absorption rate of cholesterol, which ranges from 35% to 70%. As a result, around 95% of the phytosterols consumed are excreted without being absorbed. After consumption, phytosterols combine with products from triglyceride breakdown, bile acids, lecithin, and other nutrients to create micelles. In the intestine, phytosterols compete with cholesterol for absorption through the NPC1L1 transporter and are taken up by intestinal epithelial cells. Once inside these cells, free phytosterols are esterified by acyl-coenzyme A, transforming them into chylomicrons that contain triacylglycerol and apolipoprotein B48. On the other hand, unesterified phytosterols are sent back into the intestinal lumen via ATP-binding cassette transporters ABCG5/G8, where they can be metabolized by gut microbiota into coprostanol and coprostanone [115,116].

To significantly improve the absorption and bioavailability of phytosterols, structural modifications are often required. Phytosterols typically have high melting points and low solubility, which restricts their bioavailability. However, when they are esterified with medium- or long-chain fatty acids, their physicochemical properties can change, allowing them to blend more effectively into fat-containing food matrices and thus enhancing their bioavailability. For example, one study found that phytosterol linoleate has a bioavailability of 4.93%, which is 2.5 times greater than that of unmodified phytosterols [117]. Another method for boosting phytosterol bioavailability is microencapsulation. By utilizing nanoparticles that have improved adhesion in the gastrointestinal tract, the time they spend in digestion is extended, leading to increased absorption rates. However, excessive accumulation of phytosterols in plasma, adipose tissue, skin, the aorta, and other organs may lead to premature coronary artery disease, xanthomatosis, and early-onset atherosclerosis. Clinical evidence has shown that pediatric patients with intestinal failure who received parenteral phytosterols exhibited hepatic accumulation, which was linked to biochemical liver damage, portal inflammation, and hepatic fibrosis [118].

## 5. Conclusions

Accurate assessment of phytosterol functionality and bioavailability requires method selection optimized for matrix complexity and analytical goals. Among extraction methods, DLLME stands out for its minimal solvent use and superior enrichment factors, allowing high sensitivity and rapid extraction—especially valuable for detecting trace phytosterols in complex food matrices. However, it demands precise solvent selection and operator skill, limiting its accessibility for routine use. MAE increases extraction efficiency through heat and pressure but may degrade thermolabile sterols, making it less suitable for delicate compounds.

On the analytical side, LC-MS/MS with APCI offers exceptional sensitivity (LOD: 0.1–0.5 ng/mL) and selectivity without the need for derivatization, making it ideal for polar, non-volatile phytosterols. Its major limitations are high instrument cost and specialized maintenance needs. In contrast, GC-MS, when paired with derivatization and SPE pretreatment, excels in separating structurally similar sterols in lipid-rich matrices like oils, offering robust resolution and reproducibility, but requires complex sample preparation and is less suitable for non-volatile compounds.

Therefore, DLLME-LC-MS/MS with APCI is preferred for sensitive, high-throughput profiling in diverse food matrices, while SPE-GC-MS remains optimal for precise quantification in oil-based systems. Selecting the most appropriate approach requires careful consideration of sensitivity, matrix compatibility, operational complexity, and cost-efficiency, all of which are critical for advancing phytosterol applications in functional foods and health-related formulations.

With the support of these advanced analytical techniques and improved extraction methods, phytosterols have been shown to reduce LDL and total cholesterol levels in different populations, thereby providing benefits for cardiovascular health. Additionally, their roles in anti-inflammatory, anticancer, antidiabetic, and neuroprotective effects are well established. However, phytosterols have very low gastrointestinal bioavailability due to their low solubility in water and oil, high crystallinity, and vulnerability to oxidation. To overcome these challenges, various chemical and physical modifications, such as esterification and nanoencapsulation, have been investigated, showing promising enhancements in solubility and absorption. Nonetheless, these methods may raise concerns about the formation of oxidative byproducts, nanoparticle toxicity, and the potential for excessive accumulation in body tissues. Consequently, future research should take a holistic approach, incorporating pre-clinical and clinical evaluations of bioavailability and safety, as well as exploring the interactions between phytosterols and food matrices. Such thorough efforts are essential for promoting the safe and effective use of phytosterols in functional foods and nutraceuticals.

## 6. Methodology

Among the extensive body of literature on phytosterols, this study rigorously selected scientific publications that addressed extraction methods, analytical techniques, and biological activities related to phytosterols—including β-sitosterol, stigmasterol, campesterol, fucosterol, brassicasterol, and ergosterol. All relevant articles published to date, regardless of publication year, were considered. The literature was collected from major scientific databases, including Scopus, Web of Science, Google Scholar, Elsevier, and PubMed. Of the 452 articles initially identified, 53 were ultimately included in the final analysis. Studies published in languages other than English and those with seemingly relevant titles but content unrelated to phytosterols were excluded.

## Figures and Tables

**Table 2:** Representative studies on the therapeutic effects of phytosterols in various disease models.

Source of Phytosterols	Models	Experimental Design	Major Findings	Conclusions	References
*Cajanus cajan* L. (pigeon pea) oil rich in phytosterols	High-fat-diet (HFD)-induced dyslipidemia in Syrian hamsters	Hamsters were fed an HFD containing 20%, 40%, and 80% *Cajanus cajan* L. oil for 8 weeks.	*C. cajan* L. oil improved lipid profiles and glucose tolerance in HFD-induced hyperlipidemic hamsters by upregulating cholesterol 7 alpha-hydroxylase (CYP7A1) and carnitine palmitoyltransferase 1 (CPT-1) expression, enhancing bile acid synthesis and β-oxidation, and reducing hepatic lipid accumulation.	*C. cajan* L. oil-derived phytosterols can modulate lipid metabolism and serve as potential therapeutic agents against metabolic disorders like hyperlipidemia and type 2 diabetes.	[89]
Phytosterol, containing 40% β-sitosterol, 20% campesterol, and over 14% stigmasterol	HFD-induced hyperlipidemia in C57BL/6J mice	Phytosterol (100 mg/kg) was administered from week 4 of a 12-week HFD.	Phytosterol improved lipid profiles, enhanced hepatic antioxidant activity, and modulated key genes, such as CYP7A1, sterol 12-alpha-hydroxylase (CYP8B1), sterol 27-hydroxylase (CYP27A1), and oxysterol 7-alpha-hydroxylase (CYP7B1). Phytosterols also regulated cholesterol metabolism pathways and modulated the composition of the gut microbiota.	Phytosterols improved antioxidant function and reduced hyperlipidemia by modulating cholesterol metabolism and shifting bile acid synthesis to the alternative pathway.	[90]
Dietary phytosterols	Clinical trials	6805 participants consumed 2.15 g of plant sterols per day for 21 to 182 days.	Phytosterol supplementation dose-dependently reduced LDL-C by ~9% at 2 g/day, with effects plateauing around 3 g/day. No significant differences were found between sterols and stanols or between esterified and free forms.	This meta-analysis supports phytosterols as an effective LDL-C-lowering strategy, with further study needed on dosing and form.	[91]
Plant-based sterol	Clinical trials	26 men consumed low-fat milk with or without 2.2 g of plant sterols (free sterols or sterol esters) for 1 week.	Both sterol-enriched milks reduced cholesterol absorption by ~60%. The bioavailability of β-carotene and α-tocopherol decreased by approximately 50% and 20%, respectively, with smaller reductions seen in the sterol-free form.	Plant sterols reduce the absorption of cholesterol, β-carotene, and α-tocopherol, with sterol esters having a stronger effect.	[92]
β-sitosterol	Oxidized-LDL(oxLDL)-induced RAW 264.7 cells	Cells were pretreated with 10 μM β-sitosterol for 3 h, then exposed to 40 μg/mL oxLDL for 30 min.	β-Sitosterol inhibited H_2_O_2_, arachidonic acid (AA), and prostaglandin E2 (PGE_2_) generation in oxLDL-treated RAW 264.7 cells. The presence of β-sitosterol enhanced the inhibitory effects of tyrosol and resveratrol on oxLDL-induced H_2_O_2_, AA, and PGE_2_ production.	The synergistic effect of β-sitosterol and polyphenol in olive oil may prevent the release of reactive oxygen species (ROS) and inflammatory mediators in oxLDL-treated macrophages.	[93]
Ergosterol, stigmasterol, β-sitosterol, campesterol, and ergosterol acetate	Lipopolysaccharide (LPS, 1 μg/mL)-stimulated RAW 264.7 cells	LPS-stimulated RAW 264.7 cells were treated with 25, 50, 100, and 200 μM of phytosterols.	Phytosterols reduced tumor necrosis factor-α (TNF-α), nitric oxide (NO), and related protein expression in LPS-stimulated macrophages, with effects linked to extracellular signal-regulated kinase (ERK) pathway regulation and structural features like C-22 double bonds and C-24 side chains.	Phytosterols have anti-inflammatory potential, warranting further research on their structure–activity relationships.	[94]
NRF21 diet	Dextran sodium sulfate (DSS)-induced colitis in Balb/c mice	Mice were fed a phytosterol-enriched diet (400 mg/kg/day) or control diet for 14 days, with 5% DSS given until day 10, which was then replaced by water.	Phytosterol supplementation alleviated DSS-induced colitis by reducing inflammation and oxidative stress, promoting mucosal healing, improving gut motility, and preserving muscle contractions. It also restored cholinergic signaling by upregulating muscarinic receptors impaired by inflammation.	Phytosterol pretreatment alleviates DSS-induced colitis through antioxidant, bile acid, and microbiota modulation, showing potential as a functional food for inflammatory bowel disease (IBD) management.	[95]
Lupeol and β-sitosterol isolated from *Paederia foetida* leaves	PC3 and DU145 prostate cancer cell	PC-3 and DU145 prostate cancer cells were treated with β-sitosterol (80 μM) and lupeol (40 μM).	Lupeol and β-sitosterol reactivated cadherin-1 (CDH1) by suppressing DNA (cytosine-5)-methyltransferase 1 (DNMT1) and histone deacetylase (HDAC), inhibited prostate cancer cell growth and migration, induced apoptosis, and reduced TNF-α expression.	Lupeol and β-sitosterol show anticancer effects in prostate cancer through epigenetic, apoptotic, and anti-inflammatory pathways, highlighting *P. foetida*’s therapeutic potential.	[96]
Stigmasterol isolated from *Azadirachta indica*	DMBA (7,12-dimethylbenz[a]anthracene)-induced skin carcinoma in Swiss albino mice	DMBA (100 μg/100 μL acetone) and 1% croton oil were applied topically, and stigmasterol (400 mg/kg) was given orally three times weekly for 16 weeks.	Stigmasterol exhibited potent anticancer and antioxidant effects by inhibiting DMBA-induced skin papillomas, reducing oxidative stress, and enhancing antioxidant enzyme activity. It also promoted tissue repair and prevented DNA damage.	Stigmasterol shows strong chemopreventive potential against DMBA-induced skin cancer in mice, supporting its use as a natural anticancer agent.	[97]
Dietary phytosterols	Clinical trials	The effects of a daily intake of approximately 322 mg of plant sterols were investigated in colorectal cancer patients.	Higher total phytosterol intake was linked to reduced colorectal cancer risk, while stigmasterol was associated with increased risk, especially in women. Subgroup analysis showed site-specific links, with stronger protective effects in younger individuals.	Total phytosterols reduced colorectal cancer risk, while stigmasterol increased it, especially in women, warranting further study.	[98]
Stigmasterol	NS-1 cells and human islets exposed to HGP (30 mM glucose, 0.5 mM palmitate)	Stigmasterol (50 μg/mL) was tested for protecting β-cells from glucolipotoxicity by reducing stress and preserving insulin function.	Stigmasterol protects pancreatic β-cells from glucolipotoxicity by reducing ROS and cholesterol, improving insulin secretion, and lowering apoptosis. Stigmasterol also restores glucose-stimulated insulin secretion in NS-1 cells by inhibiting glucose-induced actin polymerization.	Stigmasterol improves β-cell function under diabetic conditions and may help treat type 2 diabetes, pending further in vivo validation.	[99]
β-sitosterol	High-fat-diet- and high-sucrose-diet-induced type 2 diabetes in albino rats	β-Sitosterol (5, 10, 20, and 30 mg/kg) was administered from day 30 of a 60-day HFD.	β-Sitosterol improved glucose and lipid metabolism in diabetic rats by enhancing insulin signaling, increasing glucose transporter 4 (GLUT4), and reducing oxidative stress.	β-Sitosterol may help manage type 2 diabetes by improving insulin signaling and reducing oxidative stress.	[100]
Phytosterol-rich margarine spread	Clinical trials	A phytosterol-rich margarine spread (2 g phytosterols per 10 g) was consumed twice daily until late pregnancy.	In patients with gestational diabetes mellitus (GDM), daily consumption of the phytosterol-enriched spread improved maternal lipid and glucose metabolism, as evidenced by reductions in triacylglycerol (TAG), total cholesterol (TC), low-density lipoprotein (LDL), insulin levels, and the homeostatic model assessment of insulin resistance (HOMA-IR), alongside increases in high-density lipoprotein (HDL) and the quantitative insulin sensitivity check index (QUICKI). Neonatal complications, including low birth weight and hypoglycemia, were also reduced.	Phytosterol-enriched spread may improve maternal metabolic profiles and reduce neonatal complications in diabetic patients.	[101]
Fucosterol isolated from *Eisenia stolonifera*	Amyloid-β (Aβ)_1–42_-induced cognitive impairment in aged rats	Aged rats received fucosterol (10 μmol/h) for 4 weeks and Aβ_1–42_ (25 mg/kg) for 7 days to induce cognitive impairment.	Fucosterol protected against Aβ_1–42_-induced endoplasmic reticulum stress (ER) and cognitive impairment by restoring brain-derived neurotrophic factor (BDNF) expression and activating the tropomyosin receptor kinase B–extracellular signal-regulated kinase 1/2 (TrkB-ERK1/2) signaling pathway, which is essential for neuronal survival and memory function.	Fucosterol’s potential as a neuroprotective agent for age-related neurodegenerative diseases is suggested.	[102]
β-Sitosterol	AlCl_3_-induced cognitive impairment in C57BL/6 mice	AlCl_3_ (10 mg/kg) and β-sitosterol (25 mg/kg) were administered for 21 days.	β-Sitosterol improves cognitive function in Alzheimer’s models by crossing the blood–brain barrier (BBB), reducing Aβ and acetylcholinesterase (AChE) levels, enhancing memory, and boosting brain glutathione (GSH), with potential Aβ clearance via β-site amyloid precursor protein-cleaving enzyme 1 (BACE1) inhibition or secretase activation.	β-Sitosterol prevents AlCl_3_-induced cognitive impairment and shows potential for Alzheimer’s disease (AD) management through its neuroprotective, anticholinesterase, and anti-inflammatory effects, though its mechanisms require further investigation.	[103]

## Data Availability

Sharing of the published data is subject to federal and institutional policies, and a request should be made to the corresponding authors.

## Abbreviations

ABCG5/G8	ATP-binding cassette transporter G5
AChE	Acetylcholinesterase
AD	Alzheimer’s disease
APP	Amyloid precursor protein
Aβ	Amyloid-β
BACE1	β-Site amyloid precursor protein-cleaving enzyme 1
BBB	Blood–brain barrier
BDNF	Brain-derived neurotrophic factor
COX-2	Cyclooxygenase-2
CVDs	Cardiovascular diseases
DLLME	Dispersive liquid–liquid microextraction
DMBA	7,12-Dimethylbenz[a]anthracene
DSS	Dextran sulfate sodium
DSS	Dextran sodium sulfate
ER	Reticulum stress
ERK	Extracellular signal-regulated kinase
FPG	Fasting plasma glucose
GC	Gas chromatography
GLUT4	Glucose transporter 4
GSH	Glutathione
HDACs	Histone deacetylases
HDL	High-density lipoprotein
HOMA-IR	Homeostatic model assessment for insulin resistance
HPLC	High-performance liquid chromatography
IBD	Inflammatory bowel disease
IBD	Inflammatory bowel disease
iNOS	Inducible nitric oxide synthase
LDL-C	Low-density lipoprotein cholesterol
LLE	Liquid–liquid extraction
LLME	Liquid–liquid microextraction
LPS	Lipopolysaccharide
MAE	Microwave-assisted extraction
MAPK	Mitogen-activated protein kinase
MPO	Myeloperoxidase
NF-κB	Nuclear factor-κ B
NO	Nitric oxide
NPC1L1	Niemann-Pick C1-like 1
oxLDL	Oxidized low-density lipoprotein
PGE_2_	Prostaglandin E_2_
PLE	Pressurized liquid extraction
QUICKI	Quantitative insulin sensitivity check index
ROS	Reactive oxygen species
SFC	Supercritical fluid chromatography
SLE	Solid–liquid extraction
SPE	Solid-phase extraction
SPME	Solid-phase microextraction
TG	Triglyceride
TC	Total cholesterol
TNF-α	Tumor necrosis factor-α
TrkB-ERK1/2	Tropomyosin receptor kinase B–extracellular signal-regulated kinase 1/2
UAE	Ultrasonic-assisted extraction
